# Performance in the MRCP(UK) Examination 2003–4: analysis of pass rates of UK graduates in relation to self-declared ethnicity and gender

**DOI:** 10.1186/1741-7015-5-8

**Published:** 2007-05-03

**Authors:** Neil G Dewhurst, Chris McManus, Jennifer Mollon, Jane E Dacre, Allister J Vale

**Affiliations:** 1 MRCP(UK) Central Office, 11, St. Andrew's Place, London NW1 4LE, UK; 2 Department of Psychology, University College London, UK; 3 Academic Centre for Medical Education (ACME), University College London, UK

## Abstract

**Background:**

Male students and students from ethnic minorities have been reported to underperform in undergraduate medical examinations. We examined the effects of ethnicity and gender on pass rates in UK medical graduates sitting the Membership of the Royal Colleges of Physicians in the United Kingdom [MRCP(UK)] Examination in 2003–4.

**Methods:**

Pass rates for each part of the examination were analysed for differences between graduate groupings based on self-declared ethnicity and gender.

**Results:**

All candidates declared their gender, and 84–90% declared their ethnicity. In all three parts of the examination, white candidates performed better than other ethnic groups (*P *< 0.001). In the MRCP(UK) Part 1 and Part 2 Written Examinations, there was no significant difference in pass rate between male and female graduates, nor was there any interaction between gender and ethnicity. In the Part 2 Clinical Examination (Practical Assessment of Clinical Examination Skills, PACES), women performed better than did men (*P *< 0.001). Non-white men performed more poorly than expected, relative to white men or non-white women. Analysis of individual station marks showed significant interaction between candidate and examiner ethnicity for performance on communication skills (*P *= 0.011), but not on clinical skills (*P *= 0.176). Analysis of overall average marks showed no interaction between candidate gender and the number of assessments made by female examiners (*P *= 0.151).

**Conclusion:**

The cause of these differences is most likely to be multifactorial, but cannot be readily explained in terms of previous educational experience or differential performance on particular parts of the examination. Potential examiner prejudice, significant only in the cases where there were two non-white examiners and the candidate was non-white, might indicate different cultural interpretations of the judgements being made.

## Background

The Membership of the Royal Colleges of Physicians in the United Kingdom [MRCP(UK)] Examination is a three-part examination providing summative assessment of knowledge requirements and clinical skills necessary for trainee physicians before undertaking higher training in internal medicine and/or a medical specialty. The Part 1 and Part 2 Written Examinations are criterion-referenced, single-version, computer-marked papers. The Part 2 Clinical Examination (Practical Assessment of Clinical Skills; PACES) assesses trainees against an agreed standard of competence in all aspects of clinical consultation. It consists of 14 assessments by 10 examiners at five Stations: two communication stations [stations 2 (history-taking) and 4 (communication skills and ethics)] and three clinical skills stations [stations 1 (respiratory and abdominal systems), 3 (cardiovascular and central nervous systems) and 5 (skin, locomotor, endocrine system and eye)].

Ethnic minority and male students may underperform in undergraduate [1-4] and postgraduate medical examinations, particularly if they have graduated from non-UK medical schools [5,6]. The aim of this study was to assess effects of ethnicity and gender for UK medical graduates on pass rates in the MRCP (UK) Examination sat in the UK in 2003–4. In the Part 2 Clinical Examination (PACES) we examined the potential for interaction between ethnicity and gender of examiners and candidates.

## Methods

### Participants

Candidates volunteered gender and ethnicity using 14 ethnic categories approved by the UK Commission for Racial Equality. Candidates who did not self-declare were subsequently invited to do so by letter. Ethnicity was grouped into eight categories: Afro-Caribbean (Black-African, Black-Caribbean, and Black-Other), Asian sub-continent (Indian, Pakistani, Bangladeshi and Asian-Other), Far East (Chinese/Chinese British and Malay), Middle Eastern (Arabic and Other Middle Eastern), Mixed, White, Other and Unknown (consisting of candidates who did not declare). Examiners declared gender and ethnicity using the same categories.

### Statistical analysis

The results were analysed using SPSS software (version 13.0; SPSS Inc., Chicago, IL, USA). Analysis was performed using SPSS version 13.0. A Chi-squared test was initially employed to determine any overall differences between ethnic group categories. Logistic regression was used to test differences in pass rates by ethnic group and gender, and is reported for each part of the examination. ANOVA (repeated measures analysis of variance) was used to investigate differential performance across stations. The data were then analysed to identify any interaction of candidate and examiner ethnicity and candidate and examiner gender.

## Results

### MRCP(UK) Part 1 Examination

In total, 3650 graduates made 5711 attempts at this examination; 2671 (46.8%) were by men. Of 3650 candidates, 3272 (89.6%) declared their ethnic origin; i.e. ethnicity at 5139 of 5711 (90.0%) attempts was known. Pass rates in the eight groups are shown in Table 1. Differences between groups were highly significant (χ^2 ^= 80.94, degrees of freedom (df) = 7, *P *< 0.001). Excluding the group who had not declared ethnicity, differences were still significant (χ^2 ^= 80.77, df = 6, *P *< 0.001), with white candidates having the highest pass rate. Comparison of the white group with all others combined showed a highly significant difference (χ^2 ^= 72.81, df = 1, *P *< 0.001). Comparison of the six non-white groups showed no significant differences (χ^2 ^= 8.40, df = 5, *P *= 0.14).

Similar analysis restricted to 3100 first-attempt candidates (Table 1), showed a difference in pass rate between the eight groups (χ^2 ^= 57.39, df = 7, *P *< 0.001), a difference between the seven groups with known ethnicity (χ^2 ^= 56.95, df = 6, *P *< 0.001), and a highly significant difference between white and combined other groups (χ^2 ^= 53.15, *P *< 0.001), but no significant difference between the six non-white groups (χ^2 ^= 3.91, df = 5, *P *= 0.55).

White candidates with an overall pass rate of 50.3% [95% confidence interval (CI) 48.6–52.0%] performed significantly better than did candidates from other groups (pass rate 37.9%; 95% CI 35.7–40.1%). There were no significant differences between other groups.

Data were then analysed by logistic regression, with passing or failing as the dependent variable. Predictor variables were gender (male versus female), attempt number (linear and quadratic effects), and ethnicity (white versus non-white). Preliminary analysis of all candidates who had declared their ethnicity showed that the quadratic effect of attempt was not significant (*P *= 0.18), and it was excluded from the model. There was a highly significant linear effect of attempt (b = -0.19, Wald χ^2 ^= 82.39, *P *< 0.001), with an odds ratio of 0.8 (95% CI 0.79–0.86) for each additional attempt. There was no effect of gender (b = -0.052, Wald χ^2 ^= 0.83, df = 1, *P *= 0.36). Ethnicity was highly significant (Wald χ^2 ^= 58.70, df = 1, *P *< 0.001), with white candidates being 1.58 times (95% CI 1.41–1.78) more likely to pass.

### MRCP (UK) Part 2 Written Examination

In total, 2718 graduates made 3238 attempts, 1548 (47.8%) by men. Of 2718 candidates, 2389 (87.9%) declared ethnic origin, i.e., the ethnicity of candidates at 2811 of 3238 (86.8%) attempts was known. Table 1 shows the pass rates in the eight groups. Differences between groups were highly significant (χ^2 ^= 79.02, df = 7, *P *< 0.001). Excluding the group that had not declared ethnicity, differences were still significant (χ^2 ^= 45.23, df = 6, *P *< 0.001), with the white group having the highest pass rate. Comparison of the white group with all other groups combined showed a highly significant difference (χ^2 ^= 39.81, df = 1, *P *< 0.001). Comparison of the six non-white groups showed no significant differences between groups (χ^2 ^= 4.43, df = 5, *P *= 0.49).

A similar set of analyses restricted to 2494 first-attempt candidates (Table 1), showed a difference in pass rate between the eight groups (χ^2 ^= 45.91, df = 7, *P *< 0.001), a difference between the seven groups for whom ethnicity was known (χ^2 ^= 43.47, df = 6, *P *< 0.001), and a highly significant difference between white and combined other groups (χ^2 ^= 33.78, *P *< 0.001), but no significant difference between the five non-white groups (χ^2 ^= 7.53, df = 5, *P *= 0.18).

White candidates performed significantly better (pass rate 83.1%, 95% CI 81.4–84.8%) than candidates from other groups (pass rate 72.8%, 95% CI 69.9–75.7%). There were no significant differences between other ethnic groups.

A preliminary logistic regression of candidates who had declared their ethnicity showed that the quadratic effect of attempt was not significant (*P *= 0.680), and it was excluded from the model. There was a highly significant linear effect of attempt (b = -0.456, Wald χ^2 ^= 51.12, *P *< 0.001), with an odds ratio of 0.634 (95% CI 0.56–0.72) for each additional attempt. There was no effect of gender (b = -0.104, Wald χ^2 ^= 1.160, 1, *P *= 0.28). The ethnicity effect was highly significant (Wald χ^2 ^= 30.98, df = 1, *P *< 0.001), white candidates being 1.73 times (95% CI 1.43–2.1) more likely to pass after taking into account gender and attempt number.

### MRCP(UK) Part 2 Clinical Examination (PACES)

In total, 2353 graduates made 3008 attempts, with 1541 (51.2%) made by men. Of 2353 candidates, 1988 (84.5%) declared their ethnic origin, i.e., the ethnicity of candidates at 2528 of 3008 (84.0%) attempts is known. Table 1 shows the pass rates in the eight groups. Differences between the groups were highly significant (χ^2 ^= 82.32, df = 7, *P *< 0.001). Excluding the group that had not declared ethnicity, differences were still significant (χ^2 ^= 69.16, df = 6, *P *< 0.001), with the white group having the highest pass rate. Comparison of the white group with all other groups combined showed a highly significant difference (χ^2 ^= 61.89, df = 1, *P *< 0.001). Comparison of the five non-white groups showed no significant differences between the groups (χ^2 ^= 6.31, df = 5, *P *= 0.28).

A similar analysis restricted to the 2140 first-attempt candidates (Table 1), showed a difference in pass rate between the eight groups (χ^2 ^= 52.39, df = 7, *P *< 0.001), a difference between the seven groups for whom ethnicity was known (χ^2 ^= 51.95, df = 6, *P *< 0.001), and a highly significant difference between white and combined other groups (χ^2 ^= 45.40, *P *< 0.001), but no difference between the six non-white groups (χ^2 ^= 5.61, df = 5, *P *= 0.35).

Overall, whites (pass rate of 75.5%; 95% CI 73.5–77.5%) performed significantly better than candidates from other groups (pass rate 60.3%; 95% CI 57.0–63.6%), and there were no significant differences between other ethnic groups.

A preliminary logistic regression of all candidates who had declared ethnicity showed that the quadratic effect of attempt was not significant (*P *= 0.41), and it was therefore excluded from the model. There was no linear effect of attempt (b = 0.054, Wald χ^2 ^= 0.634, *P *= 0.426), the odds ratio being 1.055 (95% CI 0.924–1.204) for each additional attempt. There was, however, a highly significant gender effect (b = 0.527, Wald χ^2 ^= 33.77, df = 1, *P *< 0.001), with female candidates being 1.69 times (95% CI 1.42–2.02) more likely to pass than male candidates (Table 2).

The ethnicity effect was also highly significant (b = 0.679, Wald χ^2 ^= 53.97, df = 1, *P *< 0.001), with white candidates being 1.973 times (95% CI 1.65–2.37) more likely to pass after taking into account gender and attempt number. A separate analysis assessed the possibility of gender × ethnicity interaction, which was found to be significant (Wald χ^2 ^= 5.51, *P *= 0.019). Non-white male trainees performed more poorly than expected, relative to white male trainees or non-white female trainees (Table 2).

**Figure 1:**
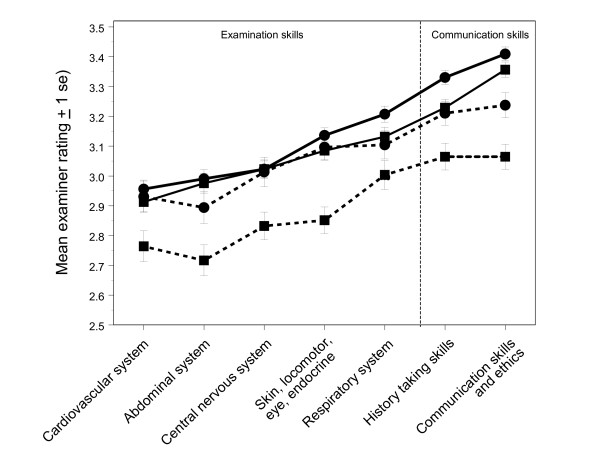
**Mean mark (± 1 SE) of candidates at each assessment, broken down by gender and ethnicity (white versus non-white) and ordered from most difficult (cardiovascular system) to least difficult (communication skills and ethics)**. The vertical dashed line separates the two communication skills stations [stations 2 (history-taking) and 4 (communication skills)] from the three clinical skills stations [stations 1 (respiratory and abdominal systems), 3 (cardiovascular and central nervous systems) and 5 (skin, locomotor, endocrine and eye)]. ■, Men; ●, women; —, white; - -, non-white.

Further analysis was undertaken to examine differential performance in each PACES station by group. For ease of interpretation, analysis was restricted to 1869 first-attempt candidates with self-declared ethnicity (classified only as white or non-white). In total, 882 (47.2%) candidates were male, 545 (29.2%) non-white, and 286 (15.3%) male and non-white. Figure 1 shows average marks received at each station (1 = clear fail; 2 = fail; 3 = pass; 4 = clear pass). Analysis was by repeated measures analysis of variance (ANOVA), with gender and ethnicity as between-subject measures, and station as a within-subject measure. Stations differed in overall difficulty (F_(6,11190) _= 68.6, *P *< 0.001). As expected from pass-rates analysis, there were also main effects of ethnicity (F_(1,1865) _= 55.5, *P *< 0.001), and gender (F_(1,1865) _= 33.0, *P *< 0.001), and a gender × ethnicity interaction (F_(1,1865) _= 10.2, *P *= 0.001). White candidates performed better overall than non-white candidates, women performed better than men (Figure 1), and the interaction indicates that non-white male candidates perform particularly poorly.

Overall, the station × ethnicity interaction was almost significant (F_(6,11190) _= 1.94, *P *= 0.071), but there was no suggestion of station × gender or station × ethnicity × gender interaction (*P *= 0.908 and *P *= 0.540 respectively). Station × ethnicity interaction was explored in a series of subanalyses. Comparison of performance on clinical skills stations with communication stations showed significant station type × ethnicity interaction (F_(1,1865_) = 4.60, *P *= 0.032). Analysis of clinical skills assessments alone showed no evidence of any interaction of clinical skills with ethnicity (*P *= 0.442) or gender (*P *= 0.772). However, analysis of the two communication stations showed significant station × ethnicity interaction (F_(1,1865) _= 3.96, *P *= 0.047), with no evidence of gender × station or gender × ethnicity × station interactions (*P *= 812 and *P *= 0.403 respectively). Inspection of Figure 1 shows that non-whites underperformed on history-taking to a similar extent to their underperformance on clinical skills, but that they also performed disproportionately poorly at the communication skills and ethics station.

As performance in PACES could depend on not only the gender and ethnicity of candidates but also on the gender and ethnicity of examiners, this aspect was analysed. Ethnicity and gender of examiners was known in 97.7% and 100% of cases respectively. Candidates are allocated at random to examiners, analysis confirming no statistical association between gender or ethnicity of candidates and examiners.

In total, 1869 first attempt candidates received a total of 2666 assessments. There were 2289 (8.8%) assessments by female examiners, with candidates having a mean of 1.23 assessments. There were 3761 assessments (14.4%) by non-white examiners, with candidates having a mean of 2.01 assessments.

Statistical analysis is complicated as the 14 assessments for each candidate are not independent. The primary analysis therefore used multiple regression to assess whether there was interaction between candidate's ethnicity (or gender) and the linear trend of the number of assessments made by non-white (or female) examiners, after taking candidate ethnicity, candidate gender, and their interaction into account. The procedure is seen most readily in Figure 1, which analyses the overall average mark (1 = clear fail; 2 = fail; 3 = pass; 4 = clear pass) of candidates according to ethnicity and number of assessments by non-white examiners. The interaction between candidate ethnicity and examiner ethnicity was almost significant (F_(1,1861) _= 3.474, *P *= 0.063), suggesting that the fitted lines in Figure 1 are probably not parallel, and that the relative difference between white and non-white candidates diminishes as the number of assessments by non-white examiners increases.

**Figure 2:**
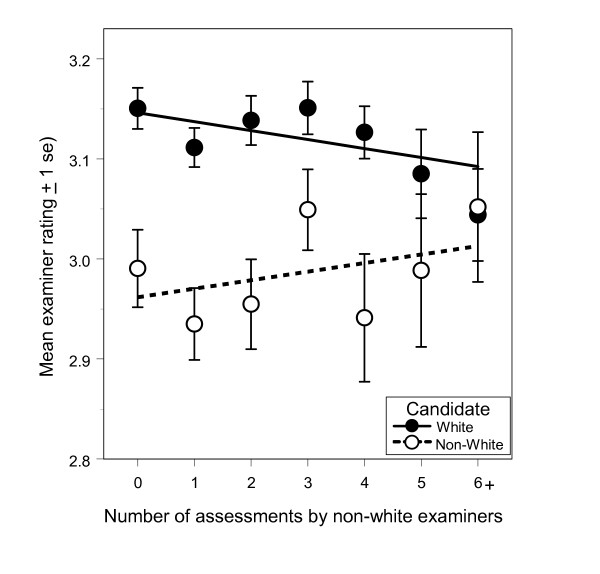
**Mean score across all 14 examiner assessments by candidate ethnicity (solid circles, white candidates; open circles, non-white candidates), in relation to the total number of assessments of a candidate that were made by non-white examiners**. Error bars indicate ± one standard error of the mean, and are wider in the right-hand points because there were fewer candidates. The solid and dashed lines indicate fitted regression lines for white and non-white candidates, weighted for different numbers of candidates.

The analysis was repeated separately for assessments made on the three clinical skills stations and the two communication stations. Interaction of examiner and candidate ethnicity was significant for the combined communication stations (F_(1861) _= 6.523, *P *= 0.011), but not for the combined clinical skills stations (F_(1861) _= 1.830, *P *= 0.176). More detailed analysis (Figure 2), shows a significant interaction of candidate and examiner ethnicity for communication skills and ethics (*p *= 0.003), a marginally significant effect for the respiratory station (*p *= 0.046), and a marginally non-significant effect for history-taking (*p *= 0.078). The largest effect on communication stations was between cases where there were two non-white examiners (non-white-non-white) and the others (white-white and white-non-white) (Figure 2). That was confirmed by showing that there was no significant interaction when analysis was restricted to cases where examiners were white-white and white-non-white (communication skills and ethics, *P *= 0.054; history-taking, *P *= 0.597; respiratory, *P *= 0.144).

Interaction of examiner and candidate gender was assessed by the statistical approach used for ethnicity. Analysis of overall average mark showed no interaction of candidate gender and the number of assessments made by female examiners (F_(1,1861) _= 2.068, *P *= 0.151). Analysis of the average mark on clinical skills stations showed no interaction between candidate gender and number of clinical skills assessments made by female examiners (F_(1,1861) _= 2.471, *P *= 0.116). Neither did average mark on communication stations show an interaction between candidate gender and the number of assessments made by female examiners (F_(1,1861) _= 0.183, *P *= 0.669).

## Discussion

Applications from non-white ethnic groups to UK medical school are increasing [7]. Relatively poor performance by ethnic minority students has been reported in the year 3 objective structured clinical examinations (OSCE) [2,4] and OSCE stations assessing communication skills in final examinations [8]. McManus *et al*. identified poorer performance by ethnic minorities across multiple assessment modalities in final examinations, concluding that differences identified could not be explained by previous examination achievement, study habits, examination style or clinical experience [1]. Male and female UK-educated Asian students, using English as their first language, performed less well than their white European peers in OSCE and written assessments [4]. An Australian study also identified poorer outcomes in finals for Indian, Asian and Middle Eastern students compared with those from Australia, New Zealand, North America and Western Europe [9]. Place of birth, schooling and preclinical undergraduate medical education could influence outcomes. At the time of data collection, we did not routinely collect data on place of birth or first language. However, as a result of updating the Colleges' policy on equality and diversity, we have recently expanded our database to include this.

Our study reveals that white candidates achieved the highest pass rates in all three parts of the MRCP(UK) Examination and it seems likely that trends already observed by others in undergraduate examinations continue through into the "high-stakes" postgraduate arena. The hypothesis that poorer achievement results from either overt or covert discrimination by examiners cannot be sustained for the MRCP(UK) Written Examinations, which are computer-marked multiple-choice papers.

One possible explanation may be that cultural differences in the perceived status of a medical career have resulted in non-white candidates making exceptional efforts to gain entrance into medical school – efforts that were unsustainable in the long term, resulting in regression to the mean. Another possibility is that for cultural reasons the best of the non-white graduates were attracted to specialties not requiring MRCP(UK), such as surgery or psychiatry, while medicine attracted the best of the white candidates. Further research looking at other postgraduate examinations would be needed to substantiate this.

Undergraduate examination success is more likely for female students [10], and although there were no overall gender differences in pass rates in the written examinations, women performed significantly better in PACES. In North American Clinical Skills Assessments, Rothman *et al *[11] found significant gender differences in 9 of 23 clinical skills stations; in 8, these differences favoured women, and similar differences have also been identified in the Educational Commission for Foreign Medical Graduates' Clinical Skills Assessments [12]. In a communication skills OSCE-style assessment in general practice, women performed better, [13] which could be related to specific traits including "the ability to listen" [14] and a greater sense of "patient care values" [15]. In addition, female practitioners may find it easier to develop co-operative approaches to doctor-patient interactions [16]. Thus, it seems probable that in any postgraduate medical examination, female candidates will perform better at assessments involving consultation and communication.

Analysis of overall average marks showed no interaction between candidate gender and the number of assessments made by female examiners, in keeping with the analysis by Ringdahl et al, which failed to demonstrate gender bias from senior residents and faculty members in rating family-practice interns [17].

Although female candidates performed better on PACES as a whole, there was no evidence that they performed particularly well on communication; rather they performed better to an equal extent on all stations. Likewise, non-white candidates performed relatively poorly on both examination skills and communication, with the sole exception that they performed particularly poorly on the communication skills and ethics station. This differential performance between ethnic minority UK graduates and white UK graduates has also been identified in PACES revision courses [18].

Performance of non-white male trainees was particularly poor across all sections of the examination. This cannot be explained readily in terms of generally poorer communicative ability, as their relative performance on the history-taking station was equivalent to that in clinical skills stations. As all candidates in this study graduated in the UK, the command and comprehension of English should not be a factor. The relative underperformance on the communication skills and ethics station may represent, however, a specific problem of cross-cultural interpretation or understanding.

Clinical examinations generate much interest in examiner fairness. In PACES, individual examiner bias is minimised by using objective rather than subjective criteria ("anchor statements") offering candidates of both sexes equal opportunity to demonstrate competence. Examiners are advised to follow the same line of questioning for each candidate-surrogate interaction minimising any potential for bias in individual encounters.

A review of MRCP(UK) examiner performance has shown non-white examiners to have a higher stringency score [19], but analysis of the joint effect of examiner ethnicity and candidate ethnicity shows a significant interaction. More detailed analysis shows that the effect is primarily occurring in the "talking stations", and there is no evidence of interaction on clinical skills stations. Any simplistic explanation in terms of examiner prejudice can be excluded, as bias would also be expected to be evident in clinical skills stations. The effect is statistically significant in the communication stations, but only, it seems, in cases where two non-white examiners meet a non-white candidate. This might reflect different cultural interpretations of judgements being made, particularly when communication skills and ethics are being assessed.

Roberts et al highlighted the problems for ethnic minority candidates in a conventional oral examination in the MRCGP examination. They postulated that candidates' styles of communication could be at odds with that of white examiners, with examiners switching between styles of discourse, leading to the potential for misunderstandings [20]. Thus, when two non-white examiners encounter a non-white candidate, the style of discourse may be more consistent, resulting in an opportunity for inadvertent positive bias.

## Conclusion

Our study has identified significant variations in pass rates for UK graduates based on their self-declared ethnicity and, in the clinical examination, gender. The cause of these differences is most likely to be multifactorial, but cannot be readily explained in terms of previous educational experience or in terms of differential performance on particular parts of the examination.

Taken overall, these detailed analyses suggest that any effects of examiner and candidate concordance or discordance of ethnicity are very small and restricted to a subset of the communication stations, and are absent on clinical skills stations. That the effect of ethnicity is not primarily an effect of bias is supported by the presence of a similar size of effect in the computer-marked Part 1 and Part 2 Written Examinations. The reasons for a significant joint effect of examiner ethnicity and candidate ethnicity are not clear, but are unlikely to include conscious or unconscious bias on the part of examiners. The findings merit a more detailed analysis of station score, candidate and examiner ethnicity and scenario topic and content. When communication skills and ethics are being assessed, different cultural interpretations may be made.

## Competing interests

The authors declare that they have no competing interests.

## Authors' contributions

ICM was responsible for data analysis, NGD and JAV produced the first draft of the paper and all authors contributed to the writing of the final manuscript.

## Pre-publication history

The pre-publication history for this paper can be accessed here:



## Figures and Tables

**Figure 3:**
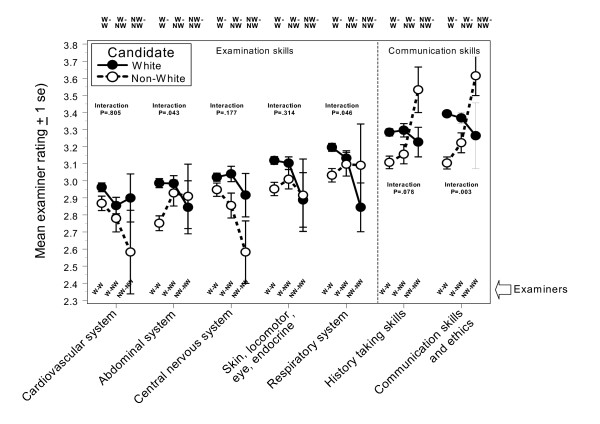
**Mean score of candidates on the assessments made at each of the five stations by candidate ethnicity (solid circles, white candidates; open circles, non-white candidates), in relation to the ethnicity of the two examiners (white-white, both examiners white; white-non-white, one examiner white and the other non-white; non-white-non-white, both examiners non-white)**. Error bars indicate ± one standard error of the mean, and are wider for the white-non-white and particularly for the non-white-non-white groups, as there were fewer candidates in those groups).

**Table 1:** Pass rates in the MRCP (UK) Examination by ethnic group

Ethnic group	MRCP (UK) Part 1 Examination		MRCP (UK) Part 2 Written Examination		MRCP (UK) Part 2 Clinical Examination*	
	
	Overall pass rate	First attempt	Overall pass rate	First attempt	Overall pass rate	First attempt
Afro-Caribbean	38.5% (30/78)	45.9% (17/37)	71.4% (30/42)	75.0% (18/24)	56.5% (26/46)	40.9% (9/22)
Asian Sub-Continent	38.5% (406/1055)	41.6% (216/519)	75.4% (370/491)	78.8% (297/377)	61.8% (280/453)	61.7% (190/308)
Far East	34.7% (174/501)	39.5% (94/238)	69.0% (167/242)	68.2% (118/173)	54.2% (110/203)	56.5% (74/131)
Middle Eastern	34.0% (32/94)	34.1% (14/41)	70.8% (34/48)	75.0% (27/36)	65.1% (28/43)	61.5% (16/26)
Mixed	46.5% (47/101)	49.2% (31/63)	68.7% (46/67)	75.6% (34/45)	69.8% (37/53)	68.4% (26/38)
White	50.3% (1641/3265)	56.3% (1065/1892)	83.1% (1573/1894)	85.7% (1344/1568)	75.5% (1287/1704)	75.7% (1001/1323)
Other	48.9% (22/45)	50.0% (13/26)	77.8% (21/27)	81.0% (17/21)	61.5% (16/26)	63.2% (12/19)
Unknown	44.9% (257/572)	53.5% (152/284)	66.7% (285/427)	78.0% (195/250)	61.7% (296/480)	68.5% (187/273)
Total	45.7% (2609/5711)	51.7% (1602/3100)	78.0% (2526/3238)	82.2% (2050/2494)	69.1% (2080/3008)	70.8% (1515/2140)

**Table 2:** Interaction of gender and ethnicity in the pass rates of candidates taking the MRCP (UK) Part 2 Clinical Examination (Practical Assessment of Clinical Examination Skills; PACES)

	Male	Female	Total
White	72.1% (586/813)	78.7% (701/891)	75.5% (1287/1704)
Non-white	52.4% (247/471)	70.8% (250/353)	60.3% (497/824)
Total	64.9% (833/1284)	76.4% (951/1244)	70.6% (1784/2528)
